# Manipulation of domain wall dynamics in amorphous microwires through the magnetoelastic anisotropy

**DOI:** 10.1186/1556-276X-7-223

**Published:** 2012-04-18

**Authors:** Arcady Zhukov, Juan Maria Blanco, Mihail Ipatov, Alexander Chizhik, Valentina Zhukova

**Affiliations:** 1 Department of Material Physics, Chemistry Faculty, Universidad del País Vasco/Euskal Herriko Unibertsitatea (UPV/EHU), P.O. Box 1072, San Sebastián, 20080, Spain; 2 IKERBASQUE, Basque Foundation for Science, Bilbao, 48011, Spain; 3 Department of Applied Physics I, Escuela Universitaria Politecnica Donostia-San Sebastian (EUPDS), Universidad del País Vasco/Euskal Herriko Unibertsitatea (UPV/EHU), Plaza Europa 1, San Sebastián, 20018, Spain

## Abstract

We studied the effect of magnetoelastic anisotropy on domain wall (DW) dynamics and remagnetization process of magnetically bistable Fe-Co-rich microwires with metallic nucleus diameters (from 1.4 to 22 μm). We manipulated the magnetoelastic anisotropy applying the tensile stresses and changing the magnetostriction constant and strength of the internal stresses. Microwires of the same composition of metallic nucleus but with different geometries exhibit different magnetic field dependence of DW velocity with different slopes. Application of stresses resulted in decrease of the DW velocity, *v*, and DW mobility, *S*. Quite fast DW propagation (*v* until 2,500 m/s at *H* about 30 A/m) has been observed in low magnetostrictive magnetically bistable Co_56_Fe_8_Ni_10_Si_10_B_16_ microwires. Consequently, we observed certain correlation between the magnetoelastic energy and DW dynamics in microwires: decreasing the magnetoelastic energy, *K*_me_, DW velocity increases.

## Background

Recent growing interest on domain wall (DW) propagation in thin magnetic wires with submicrometric and micrometric diameter is related with proposals for prospective logic and memory devices [1,2]. In these devices, information can be encoded in the magnetic states of domains in lithographically patterned nanowires [2]. DW motion along the wires allows manipulation of the stored information. The speed at which a DW can travel in a wire has an impact on the viability of many proposed technological applications in sensing, storage, and logic operation [2]. When a DW is driven by a magnetic field, *H*, parallel to the wire axis, the maximum wall speed is found to be a function of magnetic field and the wire dimensions [1,3-7]. This propagation can be driven by magnetic fields [8] reaching velocities up to 1,000 m/s or by spin-polarized electric currents in the nanowires [9]. In fact, it is essentially important not only fast domain wall propagation itself but also controlling of domain wall pinning in thin magnetic wires. Several methods of controlling domain walls in nanowires have been reported. For example, domain walls can be introduced to nanowires at low fields by injection from a large, magnetically soft region connected to a wire end using a lithographically fabricated current carrying wire to provide local field or heating [1,2]. Domain walls have been pinned at artificially created defects in thin wires elsewhere [1,2].

Last few years studies of current- and magnetic field-driven domain wall propagation in different families of thin magnetic wires (planar and cylindrical) attracted considerable attention [1-3]. Considerable attention has been paid to achieve controllable and fast domain wall propagation in thin magnetic wires (planar and cylindrical) taking into account the possibility to use it for high-density data storage devices (magnetic random memory devices, logic devices) [1].

It is worth mentioning that extremely fast DW propagation of single domain wall at relatively low magnetic field has been reported for cylindrical glass-coated amorphous microwires with positive magnetostriction constant with typical diameters of ferromagnetic nucleus about 10 to 20 μm [3,4].

Glass-coated ferromagnetic wires exhibit unusual and interesting magnetic properties such as magnetic bistability and giant magneto-impedanc effect [3,5,6]. Magnetic bistability, observed previously in few amorphous materials, is related with single and large Barkhausen jump [3,5,7]. Such behavior observed in different wire families has been interpreted as the magnetization reversal in a single large axially magnetized domain [3,8]. From the point of view of studies of DW dynamics, amorphous glass-coated microwires with positive magnetostriction constant are unique materials allowing us to study the magnetization dynamics of a single DW in a cylindrical micrometric wire. It is commonly assumed that their domain structure is determined by the stress distribution during rapid solidification fabrication process and consists of single large axial domain with magnetization oriented axially and the external domain structure with radial magnetization at the surface [3,5,8]. The magnetization process in axial direction runs through the propagation of the single head-to-head DW. It is worth mentioning that the micromagnetic origin of rapidly moving head-to-head DW in microwires is still unclear, although there are evidences that this DW is relatively thick and has complex structure [9].

At the same time, it is commonly assumed that the preparation of glass-coated microwires involving simultaneous solidification of composite microwire consisting of ferromagnetic metallic nucleus inside the glass coating introduces considerable residual stresses inside the ferromagnetic metallic nucleus [5,10]. The strength of internal stresses is determined by the thickness of glass coating and metallic nucleus diameter. This additional magnetoelastic anisotropy affects soft magnetic properties of glass-coated microwires. Consequently, one can expect that DW dynamics should be considerably affected by this magnetoelastic anisotropy. However, until now, little attention has been paid to studies of the influence of magnetoelastic anisotropy on DW dynamics in microwires [5,11].

Therefore, the purpose of this paper is to reveal the effect of magnetoelastic anisotropy on DW propagation in amorphous magnetically bistable microwires.

## Methods

We prepared a number of amorphous Fe-Co-based glass-coated microwires with different magnetostriction constants using Taylor-Ulitovky method, as described in [3-5,8,11]. Studied microwires of Co_56_Fe_8_Ni_10_Si_10_B_16_, Co_41.7_Fe_36.4_Si_10.1_B_11.8_, Fe_55_Co_23_B_11.8_Si_10.2_, Fe_16_ Co_60_Si_11_B_13_, Fe_72.75_Co_2.25_B_15_Si_10_, and Fe_70_B_15_Si_10_C_5_ compositions of ferromagnetic nucleus have positive magnetostriction constant and diameters of metallic nucleus from 1.2 to 22 μm. It is worth mentioning that the magnetostriction constant, *λ*_s_, in system (Co_*x*_Fe_1−*x*_)_75_Si_15_B_10_ changes with *x* from −5 × 10^−6^ at *x* = 1 to *λ*_s_ ≈ 35 × 10^−6^ at *x* ≈ 0.2 Therefore, producing microwires with various Fe-Co-rich compositions, we changed the magnetostriction constant from *λ*_s_ ≈ 35 × 10^−6^ for Fe-rich compositions (Fe_72.75_Co_2.25_B_15_Si_10_ and Fe_70_B_15_Si_10_C_5_) to *λ*_s_ ≈ 10^−7^ for Co_56_Fe_8_Ni_10_Si_10_B_16_ microwire (see sample details and properties in Table 1).

**Table 1:** Sample compositions, geometrical features, and properties

**Sample composition**	**Sample number**	**Sample geometry:*ρ*ratio, metallic nucleus diameter,*d*, and total diameter,*D***	**Magnetostriction constant**^**a**^
Co_56_Fe_8_Ni_10_Si_10_B_16_	1	*ρ* ≈ 0.45, *d* ≈ 13.2 μm, *D* ≈ 29.6 μm	10^−7^
Co_41.7_Fe_36.4_Si_10.1_B_11.8_	2A 2B	*ρ* = 0.39, *d* ≈ 13.6 μm, *D* ≈ 34 μm; *ρ* ≈ 0.55, *d* ≈ 13.6 μm, *D* ≈ 24.6 μm	25 × 10^−6^
Fe_55_Co_23_B_11.8_Si_10.2_	3A 3B	*ρ* ≈ 0.44, *d* ≈ 13.2 μm, *D* = 29.6 μm; *ρ* ≈ 0.55, *d* ≈ 12.6 μm, *D* = 22.8 μm	30 × 10^−6^
Fe_16_Co_60_Si_11_B_13_	4	*ρ* ≈ 0.39, *d* ≈ 13.6 μm, *D* ≈ 34.5 μm	15 × 10^−6^
Fe_72.75_Co_2.25_B_15_Si_10_	5A 5B	*ρ* ≈ 0.14, *d* ≈ 1.4 μm, *D* ≈ 10 μm; *ρ* ≈ 0.31, *d* ≈ 2.8 μm, *D* ≈ 9 μm	35 *×* 10^−6^
Fe_70_B_15_Si_10_C_5_	6A 6B 6C 6D 6E	*ρ* ≈ 0.67, *d* ≈ 14.6 μm, *D* ≈ 21.8 μm; *ρ* ≈ 0.63, *d* ≈ 15 μm, *D* ≈ 23.8 μm; *ρ* ≈ 0.48, *d* ≈ 10.8 μm, *D* ≈ 22.5 μm; *ρ* ≈ 0.26, *d* ≈ 6 μm, *D* ≈ 23 μm; *ρ* ≈ 0.16, *d* ≈ 3 μm, *D* ≈ 18 μm	35 *×* 10^−6^
Co_67_Fe_3.85_Ni_1.45_B_11.5_Si_14.5_Mo_1.7_	7A	*ρ* ≈ 0.16, *d* ≈ 21.4 μm, *D* ≈ 26.2 μm	<10^−7^

^a^Estimated from the data on amorphous wires in [12].

Within each composition of metallic nucleus, we also produced microwires with different ratios of metallic nucleus diameter and total diameter, *D*, i.e., with different ratios *ρ* = *d*/*D*. This allowed us to control residual stresses since the strength of internal stresses is determined by *ρ* ratio [5,8]. We used simple measurement method based on the classical Sixtus-Tonks-like experiments [13] and measured DW dynamics under tensile applied stresses.

It is worth mentioning that the magnetoelastic energy, *K*_me_, is given by:

(1) $$K_{\text{me}}\approx 3/2\;\lambda_{\text{s}}\sigma$$

where *σ* = *σ*_i_ + *σ*_a_ is the total stress; *σ*_i_, the internal stresses; *σ*_a_, the applied stresses; and *λ*_*s*_, the magnetostriction constant [5,8,14,15].

In this way, we studied the effect of magnetoelastic contribution on DW dynamics controlling the magnetostriction constant, applied, and/or residual stresses.

In contrary to the classical Sixtus-Tonks experiments [16], we do not need the nucleation coils to nucleate the DW since the closure domain wall already exists. The small closure domains are created at the ends of the wire in order to decrease the stray fields [8]. Regarding experimental set-up, in order to activate DW propagation always from the other wire end in our experiment, we placed one end of the sample outside the magnetization solenoid. The microwire is placed coaxially inside of the primary and pick-up coils so that one end is inside of the primary coil. Magnetic field, *H*, is generated by solenoid applying rectangular-shaped voltage. The stresses have been applied during DW dynamic measurements. We used three pick-up coils mounted along the length of the wire, and propagating DW induces electromotive force (emf) in the coils as described in [13]. These emf sharp peaks are picked up at an oscilloscope upon passing the propagating wall.

Then, DW velocity is estimated as:

(2) $$v=\frac{l}{\Delta t}$$

where *l* is the distance between pick-up coils and Δ*t* is the time difference between the maximum in the induced emf.

In our studies, we paid attention only to linear region of *v*(*H*) corresponding to viscous DW propagation, leaving apart non-linearity at high-field region, attributed by different authors to Walker-like behavior [4,17] or multiple DW nucleation at defects [13]. Hysteresis loops have been measured using vibrating sample magnetometer.

## Results and discussion

**Figure 1:**
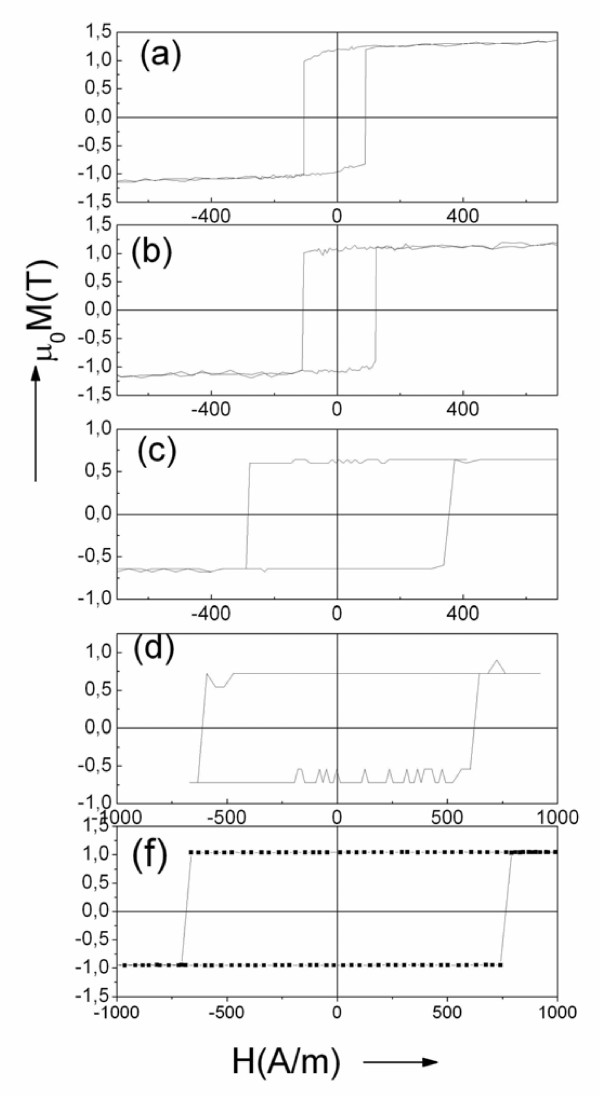
**Hysteresis loops of Fe-rich amorphous microwires.** All the samples have the same length and different metallic nucleus diameter *d* and total diameters *D*: Fe_70_B_15_Si_10_C_5_ microwires with *ρ* = 0.63, *d* = 15 μm (**a**); *μ* = 0.48, *d* = 10.8 μm (**b**); *μ* = 0.26, *d* = 6 μm (**c**); *μ* = 0.16, *d* = 3 μm (**d**); and of Fe_72.75_Co_2.25_B_15_Si_10_ microwire with *μ* = 0.14, *d* ≈ 1.4 μm, *D* ≈ 10 μm (**e**).

Hysteresis loops of a few studied microwires (Fe_70_B_15_Si_10_C_5_ and Fe_72.75_Co_2.25_B_15_Si_10_) with different metallic nucleus diameters and similar Fe-rich composition are shown in Figure 1. As can be appreciated, considerable increase of the switching field (from about 80 to 700 A/m) is observed when ferromagnetic metallic nucleus diameter decreases from 15 to 1.4 μm (i.e., one order). At the same time, rectangular hysteresis loop shape is maintained even for the smallest microwire diameters. Previously, similar increasing of coercivity with decreasing the metallic nucleus diameters has been attributed to enhanced magnetoelastic energy arising from enhanced internal stresses when *ρ* ratio is small [5,8,14]. Consequently, one of the relevant parameters affecting strength of internal stresses and the magnetoelastic energy is *ρ* ratio.

**Figure 2:**
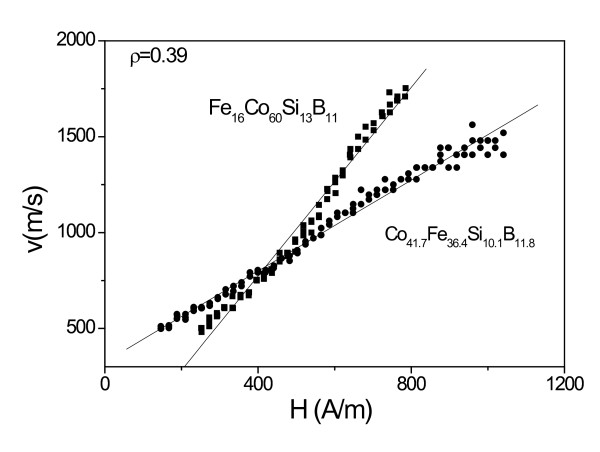
***v*(*H*) Dependences for Fe**_**16**_**Co**_**60**_**Si**_**13**_**B**_**11**_**and Co**_**41.7**_**Fe**_**36.4**_**Si**_**10.1**_**B**_**11.8**_**microwires with*ρ*** **= 0.39.**

Usually, it is assumed that domain wall propagates along the wire with a velocity:

(3) $$v=S\left(H-H_{0}\right)$$

where *S* is the DW mobility, *H* is the axial magnetic field, *H*, for Fe_16_ Co_60_Si_13_B_11_ and Co_41.7_Fe_36.4_Si_10.1_B_11.8_ amorphous microwires with the same *ρ* ratio are shown in Figure 2. In this case, the effect of only magnetostriction constant is that higher magnetostriction constant (according to [12] for Co_41.7_Fe_36.4_Si_10.1_B_11_ microwire *λ*_s_ ≈ 25 *×* 10^−6^ should be considered, while for Fe_16_Co_60_Si_13_B_11_ composition *λ*_s_ *≈* 15 × 10^*−*6^, see Table 1) results in smaller DW velocity at the same magnetic field and smaller DW mobility, *S*.

**Figure 3:**
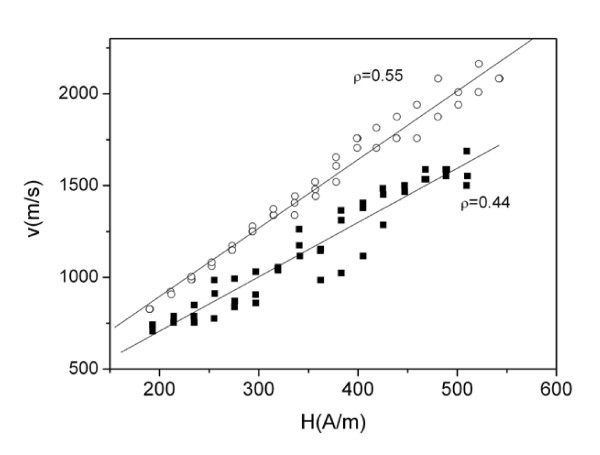
***v*(*H*) Dependences for Fe**_**55**_**Co**_**23**_**B**_**11.8**_**Si**_**10.1**_**microwires with different*ρ*ratios.**

In order to evaluate the effect of *ρ* ratio, i.e., effect of residual stresses on DW dynamics, we performed measurements of *v*(*H*) dependences in the microwires with the same composition but with different *ρ* ratios. Dependences of DW velocity on the applied field for Fe_55_Co_23_B_11.8_Si_10.1_ microwires with different ratios are shown in Figure 3. Like in Figure 2, at the same values of applied field, *H*, the domain wall velocity is higher for microwires with higher *ρ* ratio, i.e., when the internal stresses are lower [5,18].

**Figure 4:**
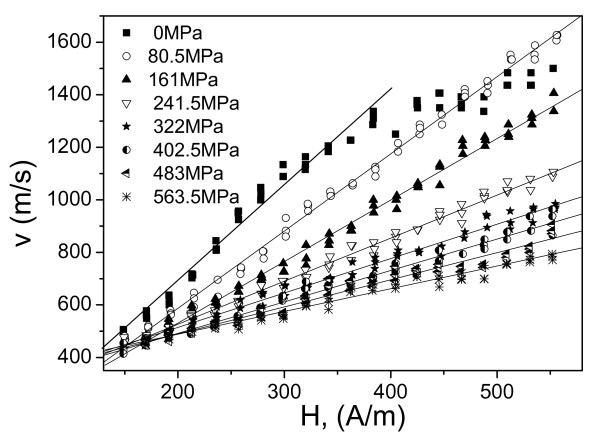
***v*(*H*) Dependences for Co**_**41.7**_**Fe**_**36.4**_**Si**_**10.1**_**B**_**11.8**_**microwires (*d*** **≈ 13.6 μm,*D*** **≈ 24.6 μm,*ρ*** **≈ 0.55).** These dependences are measured under applied stresses, *σ*_a_.

The most efficient way to change *in situ* the magnetoelastic energy is to apply stresses during measurements. Therefore, to evaluate the magnetoelastic contribution, we measured *v*(*H*) dependences applying stress. In this case, stress applied to metallic nucleus has been evaluated considering stresses distribution between the metallic nucleus and in glass coating, as previously described in [19]. We measured *v*(*H*) dependences for various microwires with different magnetosriction constant, i.e., Co_41.7_Fe_36.4_Si_10.1_B_11.8_ microwire (*ρ* ≈ 0.55) and Fe_74_B_13_Si_11_C_2_ microwire (*ρ* ≈ 0.67) under applied stresses (see Figure 4 where *v*(*H*) for microwire Co_41.7_Fe_36.4_Si_10.1_B_11.8_ is shown). Considerable decreasing of domain wall velocity, *v*, at the same magnetic field value, *H*, has been observed under applied stress. Additionally, increasing of applied stress, *σ*_a_, results in decreasing of DW velocity.

**Figure 5:**
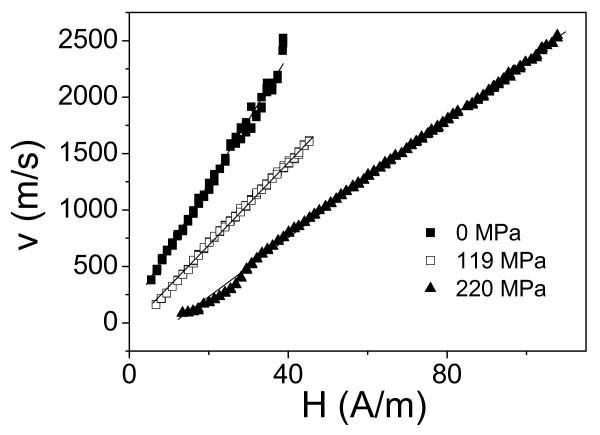
***v*(*H*) Dependences for Co**_**56**_**Fe**_**8**_**Ni**_**10**_**Si**_**10**_**B**_**16**_**microwires measured under applied stresses,*σ***_**a**_.

Finally, we measured *v*(*H*) dependences in low magnetostrictive Co_56_Fe_8_Ni_10_Si_10_B_16_ microwire. DW velocity values achieved in this microwire (see Figure 5) at the same values of applied field are considerably higher (almost twice) than those observed for microwires with higher magnetostriction constant (see Figures 2, 3, and 4).

Regarding experimentally obtained *v*(*H*) dependences shown in Figures 2, 3, 4, and 5, there are few typical features already discussed in previous publications. Thus, linear extrapolation to zero domain wall velocity (see Figures 2 and 5) gives negative values of the critical propagation field, *H*_0_. Such a negative value, previously reported for instance in [17,20], has been explained in terms of the negative nucleation field of the reversed domain. In the case of amorphous microwires, the reversed domain already exists and does not need to be nucleated by the reversed applied magnetic field. Another typical feature is non-linearity of *v*(*H*) dependences at low-field region. Such deviations from linear dependence have been previously attributed to the domain wall interaction with the distributed defects [17,20].

The domain wall dynamics in viscous regime is determined by a mobility relation (Equation 3), where *S* is the domain wall mobility given by:

(4) S=2μ0Ms/β

where *β* is the viscous damping coefficient and *μ*_0_ is the magnetic permeability of vacuum. Damping is the most relevant parameter determining the domain wall dynamics. Various contributions to viscous damping *β* have been considered, and two of them are generally accepted [4,20-22]: micro-eddy currents circulating nearby moving domain wall are the more obvious cause of damping in metals. However, the eddy current parameter *β*_e_ is considered to be negligible in high-resistive materials, like thin amorphous microwires.

The second generally accepted contribution of energy dissipation is magnetic relaxation damping, *β*_r_*,* related to a delayed rotation of electron spins. This damping is related to the Gilbert damping parameter and is inversely proportional to the domain wall width *Δ*_w_[20-22],

(5) $$\beta_{\text{r}}\approx \alpha M_{\text{s}}/\gamma \Delta \approx M_{\text{s}}{\left(K_{\text{me}}/A\right)}^{1/2}$$

where *γ* is the gyromagnetic ratio, *A* is the exchange stiffness constant, and *K*_me_ is the magnetoelastic anisotropy energy given by Equation 1.

Consequently, we can assume that the magnetoelastic energy can affect domain wall mobility, *S*, as we experimentally observed in few Co-Fe-rich microwires. Additionally, as previously shown in [23], the magnetic relaxation damping, *β*_r_*,* gives the main contribution to the total damping, *β*, when the wires are in stressed state, as the case of glass-coated microwires where glass coating induces strong internal stresses inside the metallic nucleus.

It is worth mentioning that systematic analysis of mechanisms of DW dynamics in thicker (with diameters between 30 and 120 μm) magnetostrictive amorphous wires without glass has been performed in [22] on the basis of bubble domain dynamics. The systematic analysis method in this paper is also a strong basis for considering domain propagation dynamics in glass-covered thinner magnetostrictive amorphous wires. Main assumptions on domain wall configuration in thicker wires have been performed considering that the DW length, *l*, is much more than its radius, *r (r/l* < < 10^−3^*)*. Consequently recently, we tried to extend the analysis performed in [22] to thinner glass-coated microwires (typically with diameters 10 μm) with strong internal stresses induced by the glass coating [24]. Particularly analyzing the voltage peak forms and experimental data on DW dynamics, we demonstrated that a very high DW mobility observed in magnetically bistable amorphous microwires with a diameter of about 10 μm can be associated with elongated domain shape. The experimental results can be explained in terms of the normal mobility with respect to the domain surface, which is reduced by a factor representing the domain aspect ratio estimated to be 300 for considered wire samples. On the other hand, experimental data on DW dynamics in thin microwires and analysis of the voltages on pick-up coils show that, generally, the structure of propagating DW is far from abrupt and quite complex [9,25]. Thus, the characteristic width of the head-to-head DW, *δ*, depends on many factors such as applied magnetic field, *H*: at *H* = 60 A/m, *δ* ≈ 65 d, while at *H* = 300 A/m, *δ* ≈ 40 d. Additionally, *δ* depends on magnetic anisotropy constant, *K*, being *δ*/*d* ≈ 13.5 for *K* = 10^4^ erg/cm^3^*δ*/*d* ≈ 20 for *K* = 5 × 10^3^ erg/cm^3^*δ*/*d* = 30 to 34 for *K* = 2 × 10^3^ erg/cm^3^, and *δ*/*d* = 40 to 50 for *K* = 10^3^ erg/cm^3^, respectively [25].

The numerical simulation of the head-to-head domain wall structure in nanowires with diameter *d* approximately 10 to 40 nm [26] shows that the characteristic DW width is comparable with the wire diameter, *δ*/*d* approximately 1 to 2. This is because the exchange energy contribution to the total nanowire energy dominates at very small diameters. However, with increasing of the wire diameter, the relative value of the exchange energy contribution decreases. However, for thinner wires with strong magnetoelastic anisotropy, the conditions *r/l* < < 10^−3^ considered in [22] are not realized.

**Figure 6:**
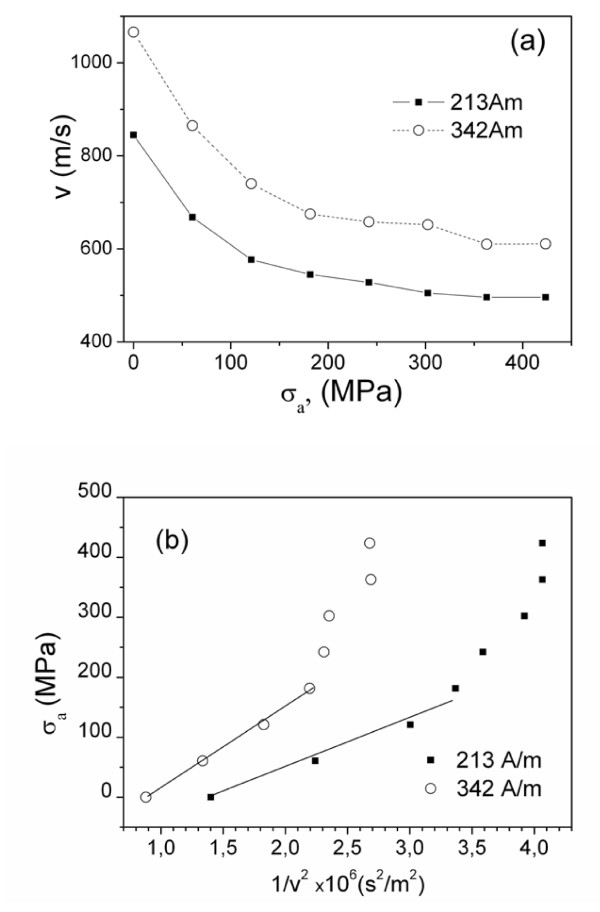
***v*(*σ***_**a**_**) Dependences of Fe**_**55**_**Co**_**23**_**B**_**11.8**_**Si**_**10.2**_**microwires.** Results for the sample 3A, *d* = 13.2 μm, *D* = 29.6 μm (**a**) and *σ*_a_(1*/v*^2^) dependence (**b**).

On the other hand, from the aforementioned, we can consider that stress dependence of DW velocity, *v*, should exhibit an inverse square root dependence. In Figures 6, we present our attempt to evaluate quantitatively observed *v*(*σ*_a_) dependence for Fe_55_Co_23_B_11.8_Si_10.2_ microwires (*d* = 13.2 μm, *D* = 29.6 μm). Experimental *v*(*σ*_*a*_) dependence exhibits decreasing of DW velocity, *v*, with applied stresses, *σ*_a_, (Figure 6a), but this dependence does not fit well with inverse square root dependence on the applied stress (Figure 6b). Here, we plotted the obtained experimentally dependences as *σ*_a_ (*v*^*−*2^). From Figure 6b, we can conclude that the obtained *v*(*σ*_a_) dependences cannot be described by single *v*(*σ*_a_^*−*1/2^) dependence. One of the possible reasons of such deviation from the predicted dependence is that when applied stresses are of the same order, as the internal stresses with complex tensor character, the effect of applied stresses on DW dynamics cannot be considered in so simple assumption. It is worth mentioning that strong internal stresses may affect magnetic properties (particularly magnetic anisotropy) of such composite materials by quite unusual way [23]. The other reason can be related with stress dependence of magnetostriction previously observed in various amorphous alloys [27]. Additionally, applied stress affects electrical resistance and, consequently, can also affect the eddy current parameter *β*_e_[28]*.*

**Figure 7:**
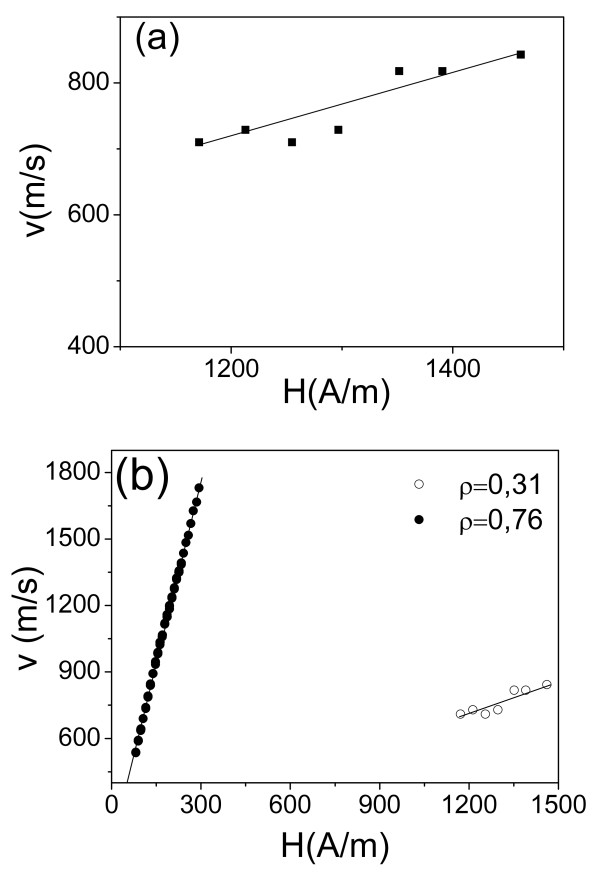
***v*(*H*) Dependence for Fe**_**72.75**_**Co**_**2.25**_**B**_**15**_**Si**_**10**_**amorphous microwire.** Results for the sample 5B with metallic nucleus diameter, *d*, of 2.8 μm and total diameter *D* ≈ 9 μm (**a**) and comparison of *v*(*H*) dependence for Fe_72.75_Co_2.25_B_15_Si_10_ amorphous microwires with different *ρ* ratios (**b**).

Regarding the aforementioned, it is interesting to compare the velocity of DW propagation in the thinnest microwire with the values observed in submicrometric planar nanowires reported elsewhere [29]. The DW velocity in thin microwire is ranging between 700 and 850 m/s (Figure 7a), which is still higher than for the same range of magnetic field as compared with submicrometric nanowires (maximum *v* ≈ 110 m/s at 700 A/m) reported elsewhere [29].

Indeed, at least in Fe_72.75_Co_2.25_B_15_Si_10_ amorphous microwire with a metallic nucleus diameter of 2.8 μm, we were able to observe domain wall propagation by the above described (Sixtus-Tonks-like) method. For such elevated magnetic fields (13 to 20 Oe), the domain wall velocity, *v*, is significantly lower than for thicker wires. For comparison, *v*(*H*) dependence for Fe_74_Si_11_B_13_C_2_ microwire with similar composition with metallic nucleus *d* and total *D* diameters 12.0/15.8 is presented in Figure 7b. As can be deduced from comparison of DW dynamics, thicker Fe_74_Si_11_B_13_C_2_ microwire at maximum achieved magnetic field (about 280 A/m) presented double higher velocity as compared with Fe_72.75_Co_2.25_B_15_Si_10_ amorphous microwire with metallic nucleus diameter, *d*, of 2.8 μm and total diameter *D* ≈ 9 μm (Figure 7b).

Regarding the observed differences on *v*(*H*) dependences, one should consider enhanced magnetoelastic energy for Fe_72.75_Co_2.25_B_15_Si_10_ amorphous microwire since ratio *ρ = d/D* determining strength of internal stresses [14,15] for thin Fe_72.75_Co_2.25_B_15_Si_10_ is *ρ* ≈ 0.31, while for thicker Fe_74_Si_11_B_13_C_2_ microwire, *ρ* ≈ 0.56.

**Figure 8:**
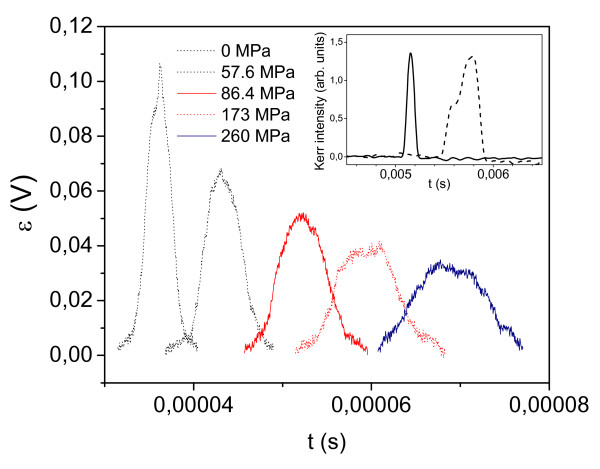
**Change of shape of the voltage from pick-up coil under tensile stress application.** Results for the sample 6A (Fe_70_B_15_Si_10_C_5_, *d* ≈ 14.6 μm, *D* ≈ 21.8 μm, *μ* ≈ 0.67). Inset, DC axial magnetic field induced transformation of the shape of the MOKE jump derivative: solid line, *H*_ax_ = 0; dashed line, *H*_ax_ = 10 A/m.

This also reflected by the change of the shape of the voltage induced in the pick-up coil surrounding microwires under tensile stress application (see Figure 8 for the Fe_74_B_13_Si_11_C_2_ microwire with *λ*_s_ *≈* 35 × 10^−6^). As-prepared microwires exhibit quite sharp voltage peaks induced in the pick-up coil associated with fast magnetization switching with the half width of the peak about 3 μs. Applying tensile stress, the half width gradually increases and at 260 MPa achieves about 8 μs. Such increasing of the half width reflects decreasing of DW velocity under tensile stress application.

Additionally, the decreasing of the DW velocity related to DW transformation has been observed when the magneto-optical Kerr effect (MOKE) modified Sixtus-Tonks method [30] has been used (see inset in Figure 8 for Co_67_Fe_3.85_Ni_1.45_B_11.5_Si_14.5_Mo_1.7_ microwire, metallic nucleus radius 10.7 μm, glass coating thickness 2.4 μm with *λ*_s_ *≈* 10^*−*7^). Application of DC axial magnetic field (*H*_ax_) additionally to the driving pulsed circular magnetic field causes the considerable transformation of the MOKE peak that in turn finds the reflection in DW deceleration. In the latter case, sharper voltage peaks, as compared with Fe-rich microwire, reflect higher DW velocity which should be attributed to lower magnetostriction constant of the Co-rich microwire.

## Conclusions

In summary, we experimentally observed that when manipulating the magnetoelastic energy through application of tensile stress and changing the magnetostriction constant and internal stresses of studied microwires, we significantly affected the domain wall dynamics in magnetically bistable microwires. Considering the aforementioned, we assume that in order to achieve higher DW propagation velocity at the same magnetic field and enhanced DW mobility, special attention should be paid to decreasing of magnetoelastic energy.

## Competing interests

The authors declare that they have no competing interests.

## Authors’ contributions

AZ provided the samples and participated in the interpretation of results. JMB measured DW propagation. MI developed the experimental setup and measured DW propagation. AC measured DW propagation by magneto-optics. VZ provided the samples and participated in the interpretation of results. All authors read and approved the final manuscript.
